# Thin Film on CMOS Active Pixel Sensor for Space Applications

**DOI:** 10.3390/s8106340

**Published:** 2008-10-13

**Authors:** Jan Dirk Schulze Spuentrup, Joachim N. Burghartz, Heinz-Gerd Graf, Christine Harendt, Franz Hutter, Markus Nicke, Uwe Schmidt, Markus Schubert, Juergen Sterzel

**Affiliations:** 1 Institut fuer Mikroelektronik Stuttgart, Allmandring 30a, 70569 Stuttgart, Germany; E-mails: burghartz@ims-chips.de; graf@ims-chips.de; harendt@ims-chips.de; hutter@ims-chips.de; markus.nicke@gmail.com; 2 Jena-Optronik GmbH, 07745 Jena, Germany; E-mails: uwe.schmidt@jena-optronik.de; juergen.sterzel@jena-optronik.de; 3 Institut fuer Physikalische Elektronik Universitaet Stuttgart / 70569 Stuttgart, Germany; E-mail: markus.schubert@ipe.uni-stuttgart.de;

**Keywords:** imager, APS, star sensor, global shutter, radiation hardness

## Abstract

A 664 × 664 element Active Pixel image Sensor (APS) with integrated analog signal processing, full frame synchronous shutter and random access for applications in star sensors is presented and discussed. A thick vertical diode array in Thin Film on CMOS (TFC) technology is explored to achieve radiation hardness and maximum fill factor.

## 1. Introduction

Autonomous star sensors determine the position of satellites and space probes by using recorded patterns of star constellations. The design of image sensors used for this application is defined by the capability of a chronological assignment and interpolation of the recorded image data with a sub-pixel resolution combined with a resistance to accumulated and temporary irradiation as well as a minimum of power consumption and weight at system level. Unlike CMOS image sensors [1] the CCD technology most commonly used on the market is less suitable because of its high demands for electronics and its lack of signal processing integration. Other CMOS products using substrate diodes [2] have a reduced capability for interpolation limited by the fill factor and the asymmetrical alignment of the photosensitive areas. Another disadvantage of this solution is a rolling shutter resulting in a distortion of dynamic images based on the time delay within a frame.

**Figure 1.**
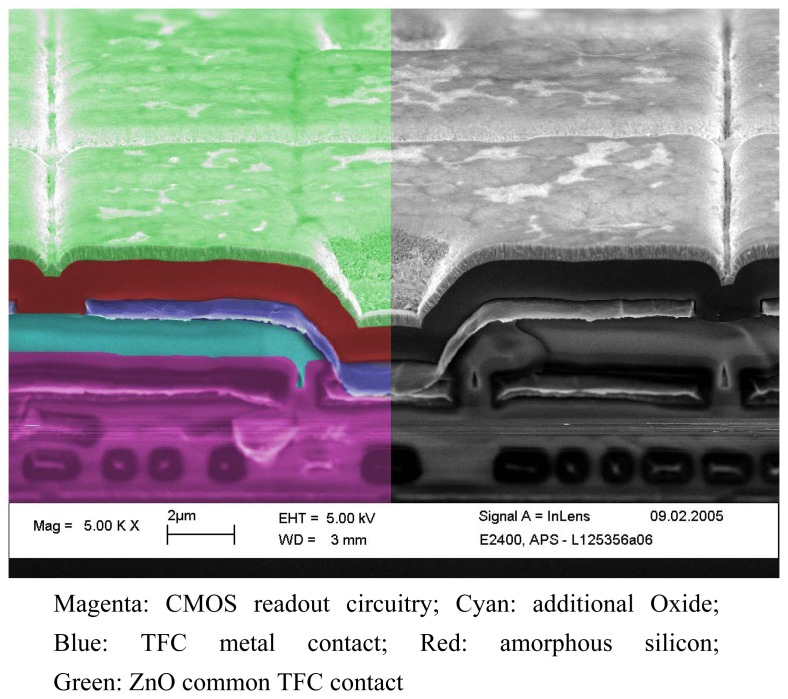
REM recording of a TFC pixel.

The active pixel imager for space applications with “Thin Film on CMOS” (TFC) technology presented here combines the advantages of an integrated signal processing with irradiation resistance and a nearly 100 % fill factor of a vertical diode array based upon the deposition of an amorphous silicon layer (see Figure 1). The low incidence of light in star sensors causes high demands on the photo diodes in terms of the quantum efficiency, dark currents, sensitivity and noise. This requires a reduced diode capacitance, which can only be achieved by amorphous vertical diodes with an α-Si layer thickness >1.7 μm. This film is much thicker than in comparable TFC sensor implementations [3-6]. The pixel with a dimension of 20 × 20 μm^2^ integrates all major functions for analog image optimization such as Correlated Double Sampling (CDS) [7] and Delta Double Sampling (DDS) by storing reset and signal data. These signal pre-processing features, normally executed in the digital part of the system are saving memory resources that are sensitive to irradiation, also reducing the number of cost- and power-intensive components. The pixel array with a resolution of 664 × 664 pixels thus enables an integrated full frame synchronous image recording (Global Shutter) with a non-destructive readout mode resulting in the ability of multi exposure acquisition and multi frame readout. The following signal path analyzes the analog signals and the CDS/DDS data of each line by using a two-phase column multiplexer with random address access. The integration of a temperature sensor and an output multiplexer for external signals completes the functionality for space applications.

## 2. Results and Discussion

### 2.1. CMOS circuit architecture

**Figure 2.**
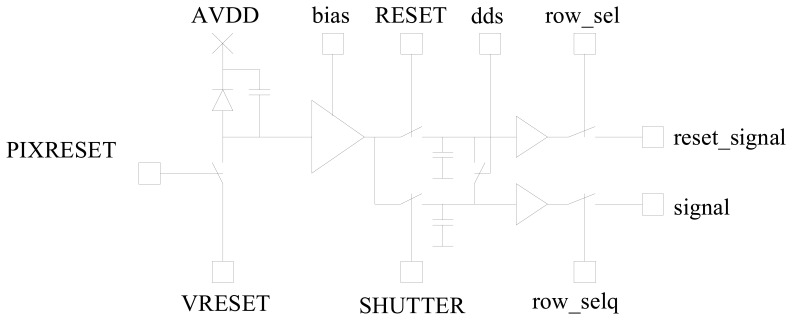
Schematic pixel design

Depending on the requirements of the application, the architecture of the APS for star sensor has an integrating pixel with a common reset and an analog preprocessing concept to minimize Reset- (kTC) and Fixed Pattern Noise (FPN) by executing a CDS and DDS to correct non-uniformities of the column multiplexers (see Figure 2). The integration time for all pixels is well controlled by an appropriate control signal timing with a pixel reset signal (*PIX_RESET*) connecting the diode node to an external reset reference voltage (*VRESET*) and a sample signal (*RESET*) storing the start value into a first capacitor and a shutter signal (*SHUTTER*) to store the light affected value into a second capacitor. Thus, the effective integration time is full field synchronous in order to realize a full field electronic shutter function. The absolute exposure time (between *RESET* and *SHUTTER*) for all detector array pixels is identical in order to allow accurate object position measurements in presence of high slew rates.

Each pixel has a non-destructive readout mode. In this case the DDS that measures the pixel output path offset with a short circuit in the pixels of one row (*dds*), is disabled. This initially allows pixel signal multiple read-outs to reduce the temporal components of the readout noise by means of averaging in the digital image processing system. Secondly, the non-destructive readout mode can be used for pixel signal monitoring during the exposure time period. This is useful for a signal dependent exposure time control, e.g. watching up-growing pixel signals up to a satisfying signal to noise ratio.

Due to the high number of signals the pixel layout is mirrored in X and Y direction enabling a share of power, reference voltages and signal lines.

**Figure 3.**
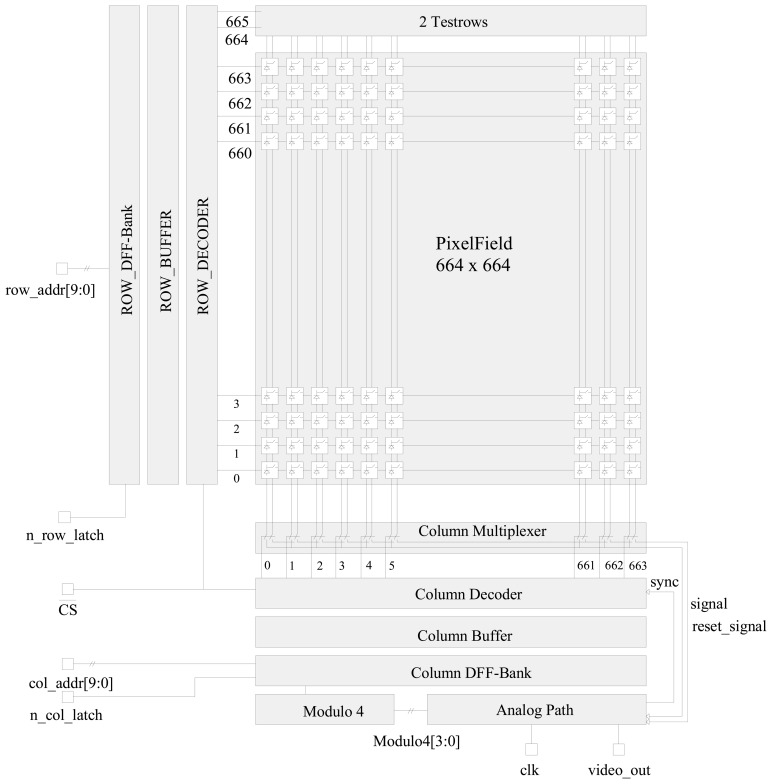
Schematic Architecture of the APS architecture

In Figure 3 the principal architecture of the detector chip is shown. Each pixel of the matrix can be individually accessed by an X and an Y address. The X address is assigned to the pixel matrix columns and the Y address is assigned to the pixel matrix rows. The address logic supports a real X/Y random access to each pixel of the pixel matrix. The address lines for the X/Y address decoder are connected separately to the device address inputs. Each address decoder is equipped with a Latch Signal Input to store the X and Y address at the same time or sequentially. The X/Y addresses are encoded in Grey-Code to avoid any peak current transitions or additional readout noise on the detector chip. The active area is quadratic and goes from 2 up to 658 (rows and columns). Additional address lines and technological structures (testrows, black covered pixels) are used for test and calibration purposes.

**Figure 4.**
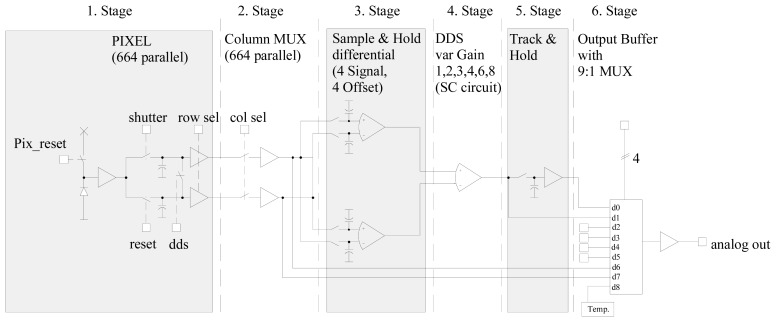
Schematic Architecture of the APS analog signal path

The analog output path in Figure 4 mainly consists of six stages. The first one is the pixel itself, with two integrated simple source follower circuits to amplify the start and the end signal values of the integration with respect to the following column multiplexer that forwards the signals to differential sample & hold circuits. At this point, there is no need for further difference building in order to execute a CDS due to the differential nature of the signals. To perform a DDS the pixel values have to be pipelined (the offset of the pixel output channel is measured with a short in each pixel of a row after two clock periods) and, therefore, 4 sample & hold stages are needed for the signals values and 4 sample & holds stages are needed for the offset values. The following stage calculates the difference between the signal and the offset values (CDS/DDS) combined with an adjustable amplification. The gain can be switched between several factors (controlled gain: 1…8 resulting in an overall gain of 2…16 of the complete analog path). Due to the SC (Switched Capacitor) nature of the DDS circuit, the output signal is just valid during a half clock period and has to be extended by a following Track & Hold stage. An analog multiplexer (1:9) allows the selection of the analog output from nine different sources. Five analog signal inputs are used for internal test signals, one for the video signal and one for an on-chip temperature sensor.

The purpose of additional analog inputs in the application field of a star sensor is to allow the possibility of processing other analog signals through the image sensor signal path. A buffer amplifier performs an analog signal conditioning with respect to the signal impedance.

A temperature sensor is placed on the detector chip for temperature monitoring purposes during the chip operation. It generates a temperature dependent voltage signal, which is led to the analog output via the analog multiplexer. The implemented linear temperature-signal characteristic is around 4 mV/ °C and has a voltage swing of around 600 mV over a temperature range of 140°C. The accuracy lies around 5% with a very good Power Supply Rejection Ratio.

**Figure 5.**
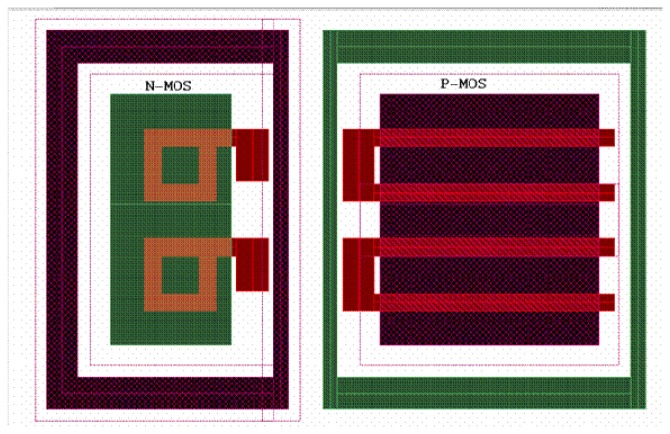
Layout schematic of the radiation tolerant design

A potential reliability problem specially associated with the CMOS circuits for space applications is *Latch Up*. This phenomenon is caused by the parasitic lateral *p-n-p* and *n-p-n* bipolar transistors created on the chip. The collectors of each transistor feed the others base, and this creates a stable “on- state” device similar to a *p-n-p-n* thyristor. This causes a sustained DC current which may cause the chip to stop functioning and may even destroy it. Technology independent methods discussed in the literature to prevent Latch Up [8, 9] were integrated during the APS for star sensor design. To realize an radiation tolerant design all critical transistors in a group of two in the digital circuitry are provided with guard rings and all NMOS transistors were arranged as ring structures (refer to figure 5). All parts of the layout were specially DRC (Design Rule Check) tested for minimized substrate- and well contact distances.

### 2.2. Thin Film on CMOS (TFC) Technology

The recognition of stars with a magnitude of 1 to 6 is necessary to measure the exact position of satellites and star probes. The accuracy can be improved by diffusing the image of the stars spots and subsequent interpolation but this also reduces the light intensity of the recorded star pattern. Therefore, high sensitivity, a wide dynamic range and a large Signal to Noise Ratio is required for this application.

Noise simulations are done splitting the analog signal path into the five stages of Figure 4 (assembling Track & Hold and Output MUX) and simulating them separately with a BSIM3v3 transistor model. This is necessary due to the switched capacitor character of the analog readout circuit. Each stage starts with the ‘hold’ circuitry of the former stage and ends with the ‘sample’ circuitry of the following stage. With respect to the output swing the first stage gain in the pixel is improved to 0.98 for noise reduction. As a result of this noise consideration a worst case input referred (to the diode node) noise of 715 μV with DDS and 620μV without DDS is calculated for 1 V photodiode voltage range.

With these worst cases simulation results the diode integration capacitance that is responsible for the transfer of the photo-generated charge can be determined. The junction capacities of the amorphous photo sensitive layer are the dominating part for this. Simulations of the photo-current and the voltage dependent diode node capacitance show the need of a 2 μm thick diode intrinsic layer. An additional 1.5 μm thick silicon oxide under the photosensitive layer reduces the influence of cross coupling from the CMOS circuitry to the photodiode node. Table 1 shows the simulation worst case (with DDS) Signal to Noise Ratios calculated with an assumed 10 bit A/D conversion of 50 dB for a mag 1 star and 19 dB for a mag 6 star with a TFC layer thickness of more than 2 μm.

The TFC system is processed in additional steps after the CMOS process and independently from the standard foundry process. The total series of deposits can be seen in Figure 1. First, an additional oxide layer (cyan layer in Figure 1) is deposited on the CMOS circuitry (magenta layer in Figure 1) to reach the desired intrinsic layer thickness. A sloped contact opening for the pixel contact is used to reduce additional leak currents in the thin film diodes caused by steep edges. The pixel metal contact area defining the photosensitive area of the pixel is formed in the next step (dark blue layer in Figure 1). According to the design rules the minimal distance between metal contacts is 1.6 μm.

The metal surface layer forms a platform for the amorphous silicon layer system (indicated red in Figure 1) deposited in subsequent low-temperature processes. Hydrogenated amorphous silicon (a-Si:H) can be deposited using plasma-enhanced CVD (Chemical Vapor Deposition) technology at temperatures between 100 and 150°C. This process has long been performed at the Institut fuer Physikalische Elektronik (Universitaet Stuttgart) [10, 11].

The deposition of the amorphous layer system consisting of the p-doped, intrinsic and n-doped layers of a-Si:H is done without breaking the vacuum. The top contact of the diodes is a transparent conductive layer, in this case zinc oxide (green layer in Figure 1). All deposition processes are carried out at temperatures well below 400 °C.

**Figure 6.**
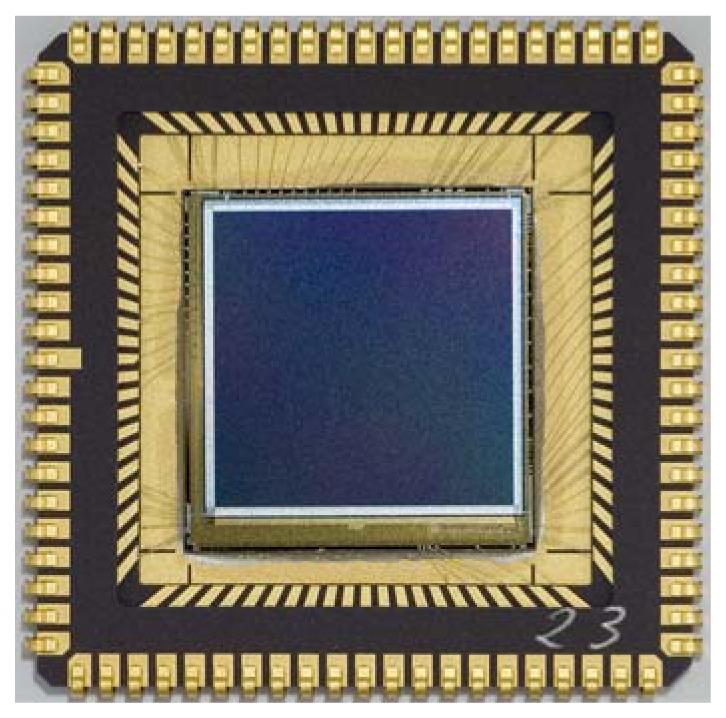
Chip Photo of the APS for star sensor with TFC layer and top contact (metal frame)

Patterning of the TFC layer system is achieved by etching. In this process only pad contacts for the CMOS chips have to be opened. The separation of pixels within the image area is achieved by the pixel metal contact and the TFC layer thus remains connected. For the top contact only one metal contact per image sensor is required, here an alumina ring (see metal frame on top of the imager device in figure 6) on top of the transparent zinc oxide that is finally protected by an antireflection layer (magnesium fluoride).

## 3. Results

The APS for star sensor was designed as an array of 664×664 elements using a 0.5 μm standard CMOS n-well process with a pixel pitch of 20 μm × 20 μm resulting in a die size of 13.3 mm × 13.3 mm (see Figure 6). PIN (in order of deposition) and NIP TFC layers were processed with different thicknesses. The top electrode was realized as an alumina frame on top of the α–Si and is connected to AVDD (NIP-layer) or AVSS (PIN-layer). The APS was designed to meet the main specification parameters that are listed in table 2.

### 3.1. Characterization of the CMOS circuitry without TFC layer

An evaluation board with a 12-bit A/D conversion and appropriate measurement software was developed to fully characterize the APS for star sensor. The deposition of a silicon oxide with a top metal layer (AL) instead of the photosensitive TFC layer enables the characterization of the CMOS readout circuitry separately from light and electrical influences from the photosensitive coating but with the same capacitance (“Simulation Mode”). Connecting the *RESET*-Voltage to a rising waveform between *RESET* and *SHUTTER* “simulates” a light response of the diodes. With this method the optical parameters of the TFC layer could be separated from the parameters of the CMOS readout circuitry.

In order to optimize the thickness of the TFC layer the temporal electronic noise had to be characterized (refer to Section 2.1). To eliminate the noise part of the evaluation board system two frames were captured with a reference voltage connected to the output path instead of the APS. The same method was rerun with different gains of the adjustable amplifier and with different integration times to obtain the noise power of the full system with the image sensor. Equations (1)–(3) show the calculation of the APS noise including the elimination of the system part.

(1) $$\begin{array}{cc} \text{difference of the pixel}\;(\text{Px}): & {\text{Diff}}_{X}=P_{\text{Frame}1X}\;-\left(\frac{P_{\text{Frame}1\;X}+P_{\text{Frame}2\;X}}{2}\right) \end{array}$$

(2) $$\begin{array}{cc} \text{noise rms}: & \text{rms}=\sqrt{2}*\sigma \left(\sum \;{\text{Diff}}_{X}\right) \end{array}$$

(3) $$\begin{array}{cc} \text{elimination of the system noise}: & {\text{rms}}_{\text{clean}}=\left(\sqrt{{\text{rms}}^{2}-{\text{rms}}_{\text{Board}}^{2}}\right) \end{array}$$

As a result of these measurements the noise is nearly constant over the integration time. It has a value of 2mV at the analog output with a default gain of 2. Calculating back the noise to the diode node using the total gain of the analog path (default: 4) results in a CMOS circuitry noise of 500 μV (DDS enabled). With these measurement results the noise specification of 100 e^-^ can already be achieved with an intrinsic- α-Si-thickness of around 1.7 μm.

To measure the linearity of the CMOS circuitry the “Simulation Mode” was used and a saw tooth voltage was connected to the top metal sublimation layer. This corresponds to a constant variation of simulated light intensities. For all varied gains (2…8) of the adjustable amplifier the linearity was within the 5% value of the specification.

The dynamic behaviour of the imager is also a very important parameter. The transition from a bright to a dark illumination in a pixel has to be fast to enable precisely tracking of star spots during satellite rotations. To separate the dynamic behaviour of the CMOS circuitry (including the diode capacitance) from the optical one of the TFC system, a 20 × 20 pixel field of an APS with a metal sublimation layer (“Simulation Mode”) was analyzed during a *RESET* voltage drop of 900 mV (simulating a transition from dark to bright and back to dark illumination). The analyzed data prove an achievement of nearly 100% of the bright and 99.75% of the dark value within a 20 × 20 pixel frame.

### 3.2. Measurements of the TFC layer

**Figure 7.**
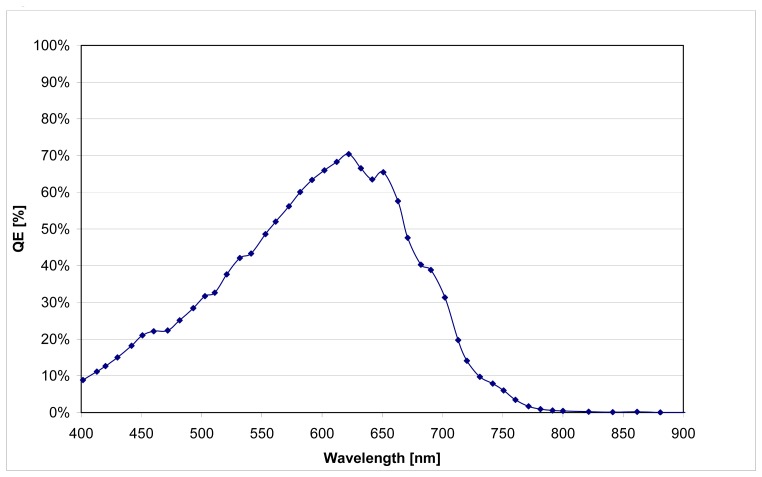
Quantum efficiency of a 800nm thick TFC PIN-diode. Diode area = 0.9 mm^2^ @ 2V rev. Bias.

The originally intended PIN (order of deposition) TFC diode structure was first realized on the CMOS based upon former work in thicknesses of 600 nm - 800 nm. After verification of the functionality, the TFC layer depositions in intrinsic layer thicknesses of 1.5 μm - 2 μm were realized to meet the specified parameters. The measurement results of the spectral sensitivity of the 800 nm show the expected behavior (see Figure 7).

A dark current of less than 70 pA/cm^2^ could be measured for both PIN TFC thicknesses and lies within the specified value of 80 pA/cm^2^. The dynamic behavior, i.e. the rise and fall time of the PIN-Diode with a periodically chopped laser flash did not show any manifestation of semiconductor inherent charge accumulation. Rise and fall times were only depending on the RC constant of the test environment. A different measurement method with strong light pulses (red Bright-LED) after a very long dark storage of the DUTs shows a good dynamic response over the first 5 decades of light but a very slow approach (up to 3 minutes) to the dark current level. This dynamic behavior is sufficient for the specified dynamic range but the required sensitivity of the APS for star sensor is not achievable with a PIN-diode TFC structure.

**Figure 8.**
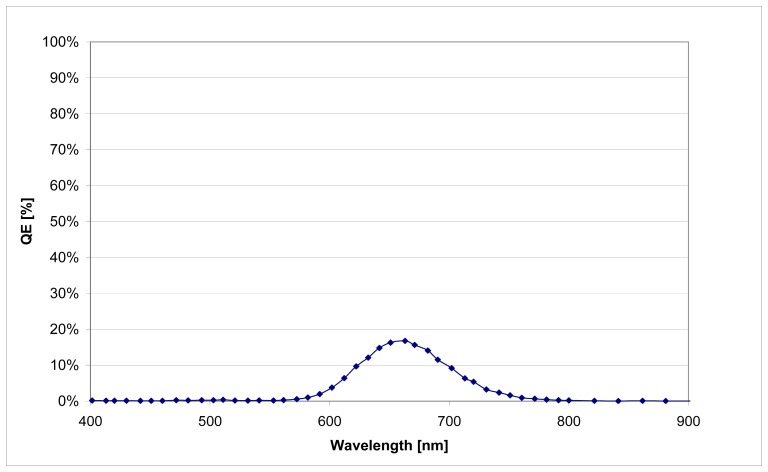
Quantum efficiency of a 2μm thick TFC PIN-diode. Diode area = 0.9 mm^2^ @ 2V rev. Bias.

**Figure 9.**
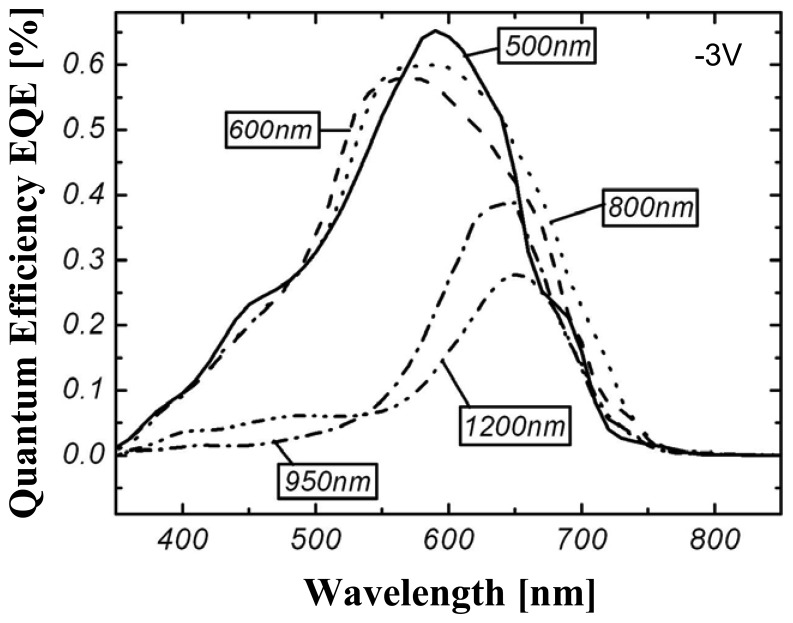
TFC Simulation Results wit ASA for different PIN diode thicknesses

Though TFC diodes with a layer thickness of 1.5 μm - 2 μm show a considerable lower spectral sensitivity (refer to Figure 8). Simulations with ASA [12] (Figure 9) prove that this effect is caused by the different mobility of the photo generated charge carriers.

**Figure 10.**
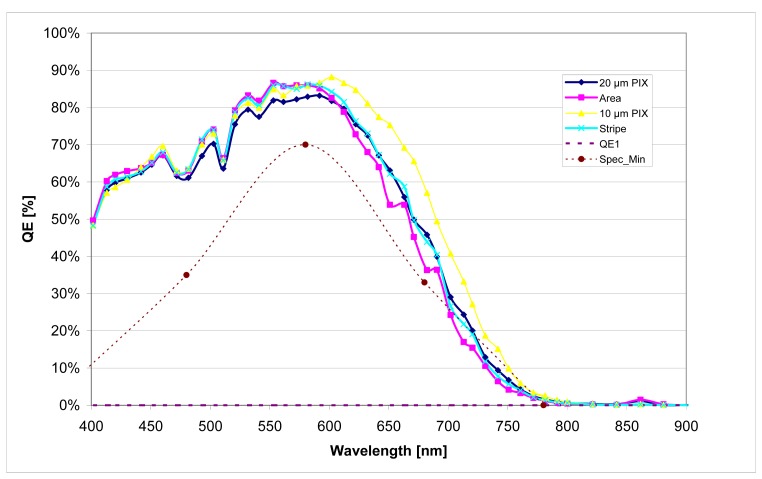
Quantum efficiency of a 1.5 μm thick TFC NIP-diode in relation to the diode structure. Diode area = 0.9 mm^2^ @ 2 V rev. Bias

The use of a NIP TFC diode for the APS for star sensor was realized with a changed deposition order sequence, adapted masks and a change of the RESET/ SHUTTER timing in the CMOS circuitry. Figure 10 shows the measured NIP-diode spectral response of a TFC layer with a thickness of 1.5 μm. It is obvious that this response meets the specification. A dark current of less then 70 pA/cm^2^ was also achieved for the 1.5 μm NIP TFC system, lying within the specification.

**Figure 11.**
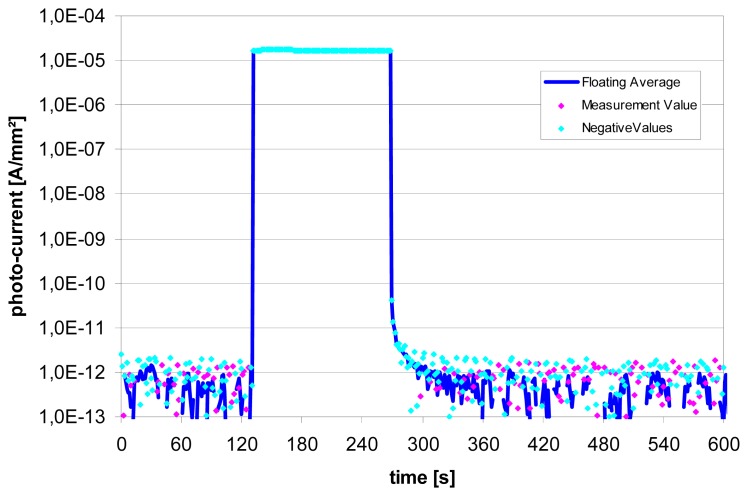
Rise- and fall time of the 1.5 μm thick TFC NIP-diode

The dynamic behavior of the NIP-diodes, measured with strong light pulses after a very long dark storage, is also better than the one of the PIN-diodes. The dark current level in this case is reached after 60s (refer to Figure 11).

### 3.3. Irradiation tests

Initially the irradiation resistance of the 0.5 μm target technology was characterized with PCM structures and a Co60 γ-irradiation source (measurement dose up to 1 MRad). The measured technology parameters of different (W/L) irradiated NMOS and PMOS transistors were compared simulation corner values that build the limits of the APS design. The results of the NMOS/ PMOS transistor measurements show the known effects. Radiation induced charges in the gate oxide change the threshold voltage and affect the leakage and saturation currents. Within an irradiation up to 100 kRad all parameters stay within their normal statistical technology variations and, thus, within design limits. An irradiation of greater than 300 kRad results in a drastically increased leakage current of the NMOS transistors. Irradiated ring oscillators did not show any variations in their frequency.

A latch up test at the GSI (Gesellschaft für Schwerionenforschung) mbH in Darmstadt was performed on a PCM test circuitry (Gold Ions with 3.6 MeV/Nucleon) and on the APS itself (tin ions with 4.8 MeV/Nucleon). The PCM had the same structure as the APS digital circuitry and neither the PCM nor the APS did show any Latch Up. During the irradiation of the APS single event upsets were detected and counted on the area of a scan chain (flip flops connected to a shift register) circuitry.

**Figure 12.**
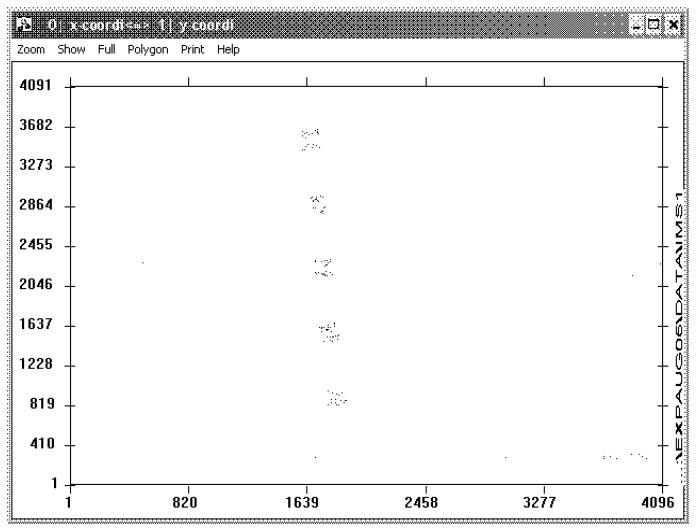
Hit image of the single event counting during irradiation with heavy ions

Figure 12 shows the hit image of the single event detection. Comparing the hits to the layout corresponds to the more sensitive PMOS transistors in the scan chain. A back calculation of the single event rate referred to the active sensor area results in a statistical value of 2875 heavy ions hitting the APS for one single event. Concluding the irradiation tests the APS for star sensor design is Latch Up free and highly resistant against irradiation. For a complete space qualification it will be necessary to perform a gamma and a proton irradiation measurement with a complete characterization of the expected optical and electrical parameter degradation during lifetime.

## 4. Conclusions

A new approach of image sensors for star sensors and space applications in general with 664 × 664 pixels in Thin Film On CMOS Technology, full frame synchronous shutter, random pixel access has been designed, fabricated and tested. The CMOS circuitry with an integrated analog signal preprocessing and a non-destructive readout mode fulfills the specification, also proving its Latch Up and irradiation resistance. A noise assessment was executed and resulted in a required TFC thickness of over 2.0 μm to meet the specification. The initially processed NIP deposition order of the α-Si was not applicable due to its drastically decreasing quantum efficiency at higher thicknesses. Based on the flexibility of the CMOS readout circuitry a reversed PIN deposition could be connected and showed a better spectral sensitivity as well as an improved dynamic performance. Devices of this type show a dark current of less than 70 pA/cm^2^ and a quantum efficiency of more than 80% for 550 nm. All specified optical and electrical parameters of the star sensor application could be achieved but unfortunately only in different TFC depositions. Further research is recommended to achieve a stable and reproducible Thin Film on CMOS process to ensure product quality and to execute the outstanding proton- and γ irradiation qualifications.

## Figures and Tables

**Table 1.** Simulation results of the TFC layer design

TFC Layer Thickness [μm]	Star Magnitude 1			Star Magnitude 6			Saturation Charge [e^-^]	Capacitance Linearity [%]

	Signal @ 0.1s integr. [e^-^]	Noise @ 0.1s integr. [e^-^]	S/N at 10 bit A/D [dB]	Signal @ 0.1s integr. [e^-^]	Noise @ 0.1s integr. [e^-^]	S/N at 10 bit A/D [dB]		

0.5	121200	498	47	1882	358	10	500000	99.2
1.0	143960	426	50	1610	199	13	272232	98.6
1.5	155220	418	50	1713	147	19	197109	98.1
1.8	159950	418	50	1759	130	19	172018	97.8
2.0	162400	419	50	1784	122	19	159575	97.6
2.2	164550	420	50	1807	115	19	149254	97.5

**Table 2.** Main APS for star sensor parameters

N°	Characteristics	Limits			Unit
		Min	Typ	Max	
1.	Signal Generation	integrating			-
2.	Shutter	full field synchronous			-
3.	Pixel Access	XY random pixel access			-
4.	Pixel Signal Read-Out	multiple non-destructive			-
5.	Power Supply	single voltage supply (+5V)			-
6.	Array Size	640 × 640			pixel
7.	Pixel Pitch	20 × 20			μm
8.	Fill Factor	85		100	%
9.	Full Well Capacity	112000		140000	e^-^
10.	Sensitivity	10 (dynamic: 1:1000)			
11.	Exposure Time	0.1		1000	ms
12.	Non Linearity up to Full Well		5	10	%
13.	Dark Signal @ 293°K			80	pA/cm^2^
14.	DSNU (1σ Full Well )			5 *	%
15.	PRNU (1σ 90% Full Well )			9 *	%
16.	Power Consumption			850	mW
17.	Signal Frequency		5	10	MHz
* With CDS and DDS									
